# The life cycle of tertiary lymphoid structures in pancreatic cancer—a window of opportunity for immunotherapy

**DOI:** 10.3389/fimmu.2026.1784463

**Published:** 2026-05-28

**Authors:** Yimin Chen, Yi Xiao, Taochun Zhang, Xinbo Wang

**Affiliations:** Research Institute of General Surgery, Jinling Hospital, Affiliated Hospital of Medical School, Nanjing University, Nanjing, China

**Keywords:** immunotherapy, neoadjuvant chemotherapy, pancreatic cancer, tertiary lymphoid structures, tumor microenvironment

## Abstract

Pancreatic cancer is a highly malignant neoplasm with a poor prognosis. The low immunogenicity of the tumor, strong immunosuppression in the tumor microenvironment (TME), and a dense tumor stroma contribute to this outcome. As a result, it is crucial to remodel the pancreatic cancer immune microenvironment to turn “cold” tumors into “hot” ones. The presence of tertiary lymphoid structures (TLS) in the TME is linked to improved prognosis and better patient outcomes. Nevertheless, the complexity of TLS biology presents several critical research gaps. Foremost, among these is the limited mechanistic understanding of how specific components of TLS--such as distinct immune cell subsets, stromal elements and their molecular interactions contribute to either beneficial or adverse clinical outcomes. Furthermore, the temporal dynamics governing TLS induction, maturation, maintenance, and resolution in response to different therapeutic modalities remain poorly elucidated. Another significant area of uncertainty pertains to the precise regulatory pathways that modulate TLS function within the immunosuppressive tumor microenvironment of pancreatic cancer. The classical maturation model of TLS describes a linear development. Based on recent advances in the evolution of TLS, we propose the concept of TLS life cycle in this review which included the tissue-resident memory phase. The mechanisms that direct TLS induction, maturation, maintenance, and resolution after various therapies are then addressed. We also discuss the identification and detection of TLS and elaborate on TLS prognostic value. We synthesize in detail the relationship between TLS and the response to therapy in PDAC. Finally, we present some preclinical evidence favoring the manipulation of TLS function and evolution within the immunosuppressive tumor microenvironment of PDAC.

## 1 Introduction

Pancreatic ductal adenocarcinoma (PDAC) is characterized by poor response to all therapeutic modalities and dismal prognosis. In recent years, scholarly attention to tertiary lymphoid structures (TLS) evolution and immunotherapeutic strategies has escalated, especially regarding their modulating tumor immune microenvironment to overcome the immunotherapy resistance in PDAC. Clinical studies underscore TLS’s prognostic value, showing that several therapies can enhance TLS evolution, suggesting potential synergy. However, gaps remain in understanding how TLS components—such as immune cell subsets, stromal elements, and their molecular interactions—mechanistically influence clinical outcomes. Additionally, detailed mechanisms underlying the regulatory pathways that modulate TLS within the immunosuppressive tumor microenvironment are not well mapped. Clarifying these interactions in this review offers new perspective for early detection, prognostication, and therapeutic innovation. The classical maturation model of TLS describes a linear development (1). Considering recent advances in the evolution of TLS, we propose the concept of TLS life cycle in PDAC which included the tissue-resident memory stage. The mechanisms that direct TLS induction, maturation, maintenance, and resolution after various therapies are then addressed. We also discuss the identification and detection of TLS and elaborate on TLS prognostic value. We synthesize in detail the relationship between TLS and the response to therapy in PDAC. Finally, we present some preclinical evidence favoring the pharmacological manipulation of TLS function and evolution within the immunosuppressive tumor microenvironment in PDAC.

## 2 Background and introduction

Pancreatic cancer is notorious for its severe malignancy and poor prognosis. Despite progress in treatments, survival remains low, with only 13% living five years after diagnosis (2). In China, the age-standardized mortality rate is 5.72/100, 000, and the incidence rate is 5.64/100, 000 (3). The incidence of pancreatic cancer is also increasing globally. The incidence of PDAC in the US is estimated to increase to 67, 440 new cases in 2025 (2). As indicated above, PDAC has the highest death rate of all major cancers estimated to reach 51, 980 deaths in 2025 (2), and it is currently the third leading cause of cancer mortality in men and women combined. Furthermore, epidemiological projections indicate that the mortality of patients with pancreatic cancer will continue to rise (4) and exceed the number of deaths from colorectal cancer by 2030, making pancreatic cancer the second leading cause of cancer-related fatalities in the US and Europe by 2030. Chemotherapy in combination with surgical resection is currently the cornerstone of treatment. The best available systemic therapies, including radiation and targeted approaches, aim to improve outcomes and modestly increase survival rates (5).

Although immunotherapy has demonstrated substantial clinical benefit in a variety of solid tumors, its efficacy in pancreatic cancer remains limited, primarily due to the tumor’s inherently low immunogenicity (6), pronounced immunosuppressive tumor microenvironment (TME) (7, 8), and dense stromal architecture (9–11). Consequently, monotherapy with immunotherapeutic agents has yielded disappointing outcomes. Recent studies have shown that the presence of TLS can improve the efficacy of chemotherapy in mouse models of pancreatic cancer (12). The combination of systemic chemotherapy (gemcitabine) and local chemokines enhance immune cell infiltration (13), fosters TLS formation, and boosts the antitumor activity of chemotherapy (14). In contrast, this promising preclinical landscape stands in stark contrast to observations from clinical cohorts. Clinical investigations in patients indicate that neoadjuvant chemotherapy (NACT) results in only minimal TLS formation and has a limited impact on overall prognosis (15). The growing evidences reveal a critical translational disconnect: why is it relatively easy to induce protective TLS in murine models, but so difficult to observe similar benefits in the clinical setting? As summarized in **Table 1**, this discrepancy raises a central question for ongoing research: can targeted modulation of TLS effectively convert immunologically “cold” pancreatic tumors into more immune responsive “hot” tumors, ultimately improving therapeutic efficacy? The following sections will examine the underlying mechanisms, translational challenges, and emerging avenues for leveraging TLS in pancreatic cancer treatment.

**Table 1:** Discrepancies in TLS induction and therapeutic outcome between preclinical models and clinical trials in pancreatic cancer.

Research type	Cancer type	Therapeutic intervention	TLS observation result	Therapeutic outcome	Ref
Preclinical Models	PDAC	systemic chemotherapy (gemcitabine)	Coadministration of systemic chemotherapy (gemcitabine) and intratumoral lymphoid chemokines into orthotopic tumors altered immune cell infiltration, facilitating TLS induction and potentiating antitumor activity of chemotherapy	This resulted in significant tumor reduction, an effect not achieved by either treatment alone. This study provides supportive evidence that TLS induction may potentiate the antitumor activity of chemotherapy in a murine model of PDAC.	(12)
Preclinical Models	PDAC	recombinant human IL-33 protein	This protein expands intratumoral lymphoneogenic ILC2s and TLS.	This protein expands intratumoral lymphoneogenic ILC2s and TLS and demonstrates enhanced anti-tumor activity in PDAC mice	(17)
Clinical Trials	PDAC	380 PDAC patients without preoperative treatment (surgery alone (SA)) and 136 patients pretreated with neoadjuvant treatment (NAT)	Within intratumoral TLSs of the NAT group, samples showed lower B-cell proportion, higher regulatory T-cell proportion, smaller size, lower maturation level, and reduced immune cell activation; moreover, the prognostic value of TLS presence was insignificant in this cohort.	the presence of intratumoral TLSs was significantly associated with improved overall survival (OS) and progression-free survival.	(15)
Clinical Trials	PDAC	nivolumab (anti-PD-1) and/or sotigalimab (CD40 agonistic antibody) with gemcitabine/nab-paclitaxel (chemotherapy)	Mature TLSs was enriched in pretreatment biopsies from PDAC patients with longer survival after receiving different chemoimmunotherapy regimens	The pre-existing mature TLS with germinal center in tumor can enhance the curative effect of CD40 agonist (sotigalimab), so as to improve the survival of patients.	(20)

ILC2s, Group 2 innate lymphoid cells; IL-33, Interleukin-33; NAT, Neoadjuvant treatment; PDAC, Pancreatic ductal adenocarcinoma; SA, Surgery alone; TLS, Tertiary lymphoid structures.

## 3 TLS and pancreatic cancer

### 3.1 Definition of lymphoid structures

The lymphoid system is divided into three groups: primary, secondary, and tertiary lymphoid organs (16). Primary lymphoid tissues, such as bone marrow and thymus, produce mature lymphocytes. Secondary lymphoid tissues, also known as secondary lymph organs (SLO), including lymph nodes, spleen, and gut-associated lymphoid tissues, help present antigens to lymphocytes. This division supports effective immune responses in the body.

TLS, also known as tertiary/ectopic lymphoid structures, are clusters of B and T cells that develop outside secondary lymphoid organs during chronic inflammation. TLS can form during inflammation or infection (17), organ transplantation (18), aging (19), and cancers (20). Structurally and functionally, TLS resemble germinal centers (GCs) to facilitate adaptive immune responses at local inflammatory sites, similar as in SLO. However, the underlying biological mechanisms driving complex multicellular structures remain poorly understood. Specifically, the distinct stages of the TLS life cycle and the signals that initiate, sustain, and terminate TLS formation warrant further study.

### 3.2 Life cycle of TLS

Our current understanding of the TLS life cycle remains incomplete and is largely based on observational studies of TLS in human tissues, as well as comparative analyses with SLO. We will review the current advances in the life cycle of TLS research from the perspective of its lymphoid neogenesis, development, maturation and tissue-resident memory processes.

#### 3.2.1 Lymphoid neogenesis of TLS

The formation of SLO is critically dependent on the crosstalk between two specialized cell types: lymphoid tissue inducer (LTi) cells and lymphoid tissue organizer (LTo) cells (21).

LTi cells are a unique subset of innate lymphoid cells characterized by the expression of the transcription factor RORγt and the inability to express typical lineage markers for T, B, or myeloid cells (22). During embryonic lymph node development, LTi cells are among the first hematopoietic cells to colonize the lymph node anlagen. Their primary function is to initiate organogenesis by clustering and activating local stromal cells (22).

Contacting with LTi cells, local mesenchymal stromal cells differentiate into LTo cells. LTo cells are defined functionally by their response to LTi-derived signals, particularly lymphotoxin (LTα1β2) (23). This interaction triggers LTo cells to upregulate adhesion molecules and key homeostatic chemokines such as CXCL13, CCL19, and CCL21 (23). This microenvironment recruiting more LTi cells and additional lymphocytes, creates a positive feedback loop leading to the compartmentalized structure of a mature lymph node (23).

Typically, TLS exhibit similarities to SLO, particularly lymph nodes, as they are compartmentalized into distinct T cell and B cell zones due to differential expression of chemokines such as CCL19, CCL21, and CXCL13 (24, 25). In addition to T cells and B cells, TLS encompasses antigen-presenting cells and structural components, including dendritic cells (DCs), follicular dendritic cells (FDCs), lymphatic vessels, and high endothelial venules (HEVs); these components are involved in the recruitment of naive lymphocytes to the sites of inflammation. Notably, GC-like structures have been identified in various TLS, including tumor-associated TLS (TA-TLS). These structures are characterized by the presence of activation-induced cytidine deaminase, FDCs, proliferating B cells, class switch recombination, somatic hypermutation, and plasma cells (26). Unlike SLO, TLS have no envelope and are transient, disappearing once the initial inducing signal is interrupted (27). This structural distinction underscores the unique role of TLS in mediating localized immune responses, particularly within chronically inflamed tissues and TME. Further investigation is needed to fully characterize the regulatory mechanisms that drive TLS formation, maintenance, and resolution, as well as their functions in pathological conditions such as pancreatic cancer.

TLS formation depends not only on immune cells but also on the surrounding tumor stroma. Fibroblast activation is a critical stage in TLS development. Fibroblasts, the most abundant non-hematopoietic stromal cells in TME, can undergo phenotypic changes under the crosstalk of tumors. CXCL12^+^COL1^+^ fibroblasts mediate the positioning of CXCR4^+^ GC and B cells and support plasma cell survival in TLS (26). Additionally, these fibroblasts facilitate the migration of early plasma cells from the GCs into chronically inflamed tissues (26). In response to inflammation, immunoblasts express adhesion molecules and secrete chemokines (25), which are critical for the recruitment and survival of immune cells, thereby playing a pivotal role in TLS formation. Once transformed into immune fibroblasts, these cells provide inducible costimulatory (ICOS) ligands, triggering two key signaling pathways essential for TLS formation: one activates ICOS-expressing lymphocytes, the other promotes lymphotoxin α3 production (28). Similar to fibroblastic reticular cells (FRCs), these immune fibroblasts form a supportive network which facilitates the recruitment and maintenance of immune cells within TLS. Meanwhile, these immune-fibroblasts exhibit characteristics of lymphoid tissue organizer cells (29, 30). Unlike in other cancer, where TLS often form at the invasive margin with direct immune access, the dense stromal barrier in PDAC may physically impede lymphocyte trafficking and TLS neogenesis. Future studies should investigate whether targeting the stroma could facilitate TLS formation.

**Figure 1:**
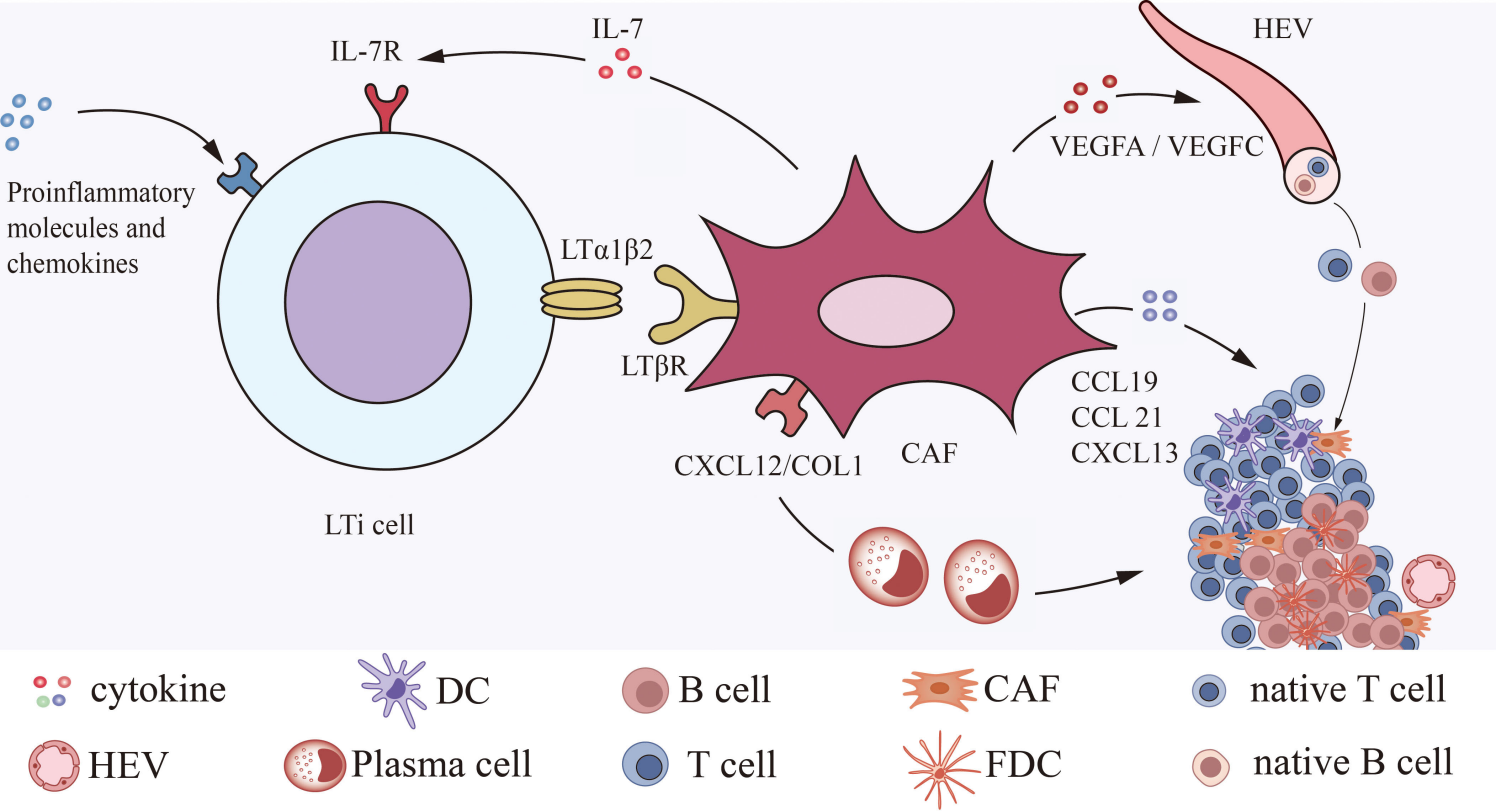
Cellular and molecular signals controlling TLS formation.The local accumulation of proinflammatory molecules and chemokines promotes the recruitment of lymphoid tissue inducer (LTi) to the site of inflammation, facilitating their interaction with stromal cells and initiating TLS formation. This process involves the expression of cytokines (IL-7, and LTα1β2) and their corresponding receptors (IL-7R and LTβR). In the absence of LTi cells, other immune cells such as macrophages, B lymphocytes, and Th17 cells can interact with stromal cells to induce TLS formation. The outcome of these interactions is the production of chemokines (CCL19, CCL21 and CXCL13), pro-angiogenic molecules (e.g., VEGF), and adhesion molecules, which collectively promote the recruitment, retention, and organization of additional immune cell types into newly generated TLS. HEV, high endothelial venules; DC, dendritic cells; FDC, follicular dendritic cells; CAF, cancer-associated fibroblast; LTi, lymphoid tissue inducer; LTβR, lymphotoxin-β receptor; LTα1β2, lymphotoxin α1β2; IL – 7, interleukin – 7; IL-7R, IL-7 receptor; VEGF, vascular endothelial growth factor; TLS:tertiary lymphoid structures.

The local accumulation of proinflammatory molecules and chemokines promotes the recruitment of LTi cells towards the inflammatory loci. This process facilitates the interaction of LTi cells and stromal cells to boost the formation of TLS (**Figure 1**). This also encompasses the production of cytokines, notably interleukin-7 (IL-7) and LTα1β2, with their receptors IL-7 receptor (IL-7R) and lymphotoxin β receptor (LTβR) (31, 32). In the absence of LTi cells, other immune cells such as macrophages, B lymphocytes and T-helper 17 (Th17) cells interact with stromal cells to promote TLS. These interactions generate chemokines such as CCL19, CCL21 and CXCL13, as well as pro-angiogenic factors such as VEGF and adhesion molecules (33). These cells interact with stromal cells, including cancer-associated fibroblasts (CAFs) and endothelial cells, primarily through the LTα1β2-LTβR and TNF-TNFR signaling axes. For instance, Th17 cells not only express LTα1β2 (34) but also secrete IL-17, which stimulates CAFs to produce chemokines (35). Similarly, M1-polarized macrophages can secrete TNF-α to activate stromal cells. These interactions generate chemokines such as CXCL13 (36), which orchestrate lymphocyte recruitment and compartmentalization, as well as pro-angiogenic factors such as VEGF and adhesion molecules (e.g., ICAM-1, VCAM-1) that facilitate HEV development and immune cell trafficking (34). These molecules together drive the recruitment, retention, and organization of more immune cell subtypes into the nascent TLS. Finally, HEVs, specialized post-capillary venules, express peripheral lymph node vascular addressins (PNAds) and ligands for L-selectin and facilitate immune cell homing into TLS (37). HEVs in TA-TLS contained extravasated various T cell subsets, suggesting that they may serve as a route for lymphocyte infiltration into tumors (38). However, the vasculature supporting TLS is heterogeneous. Recent findings in human breast cancer indicate that PNAd (MECA-79)-negative blood vessels, which express sialyl Lewis X, can also effectively recruit lymphocytes and support TLS formation, pointing to alternative, non-classical pathways for lymphocyte homing (39).

These interactions between immune cells and stromal components emphasize the complexity of TLS formation and highlight the potential of these components as therapeutic targets in cancer immunotherapy.

#### 3.2.2 Development of TLS

**Figure 2:**
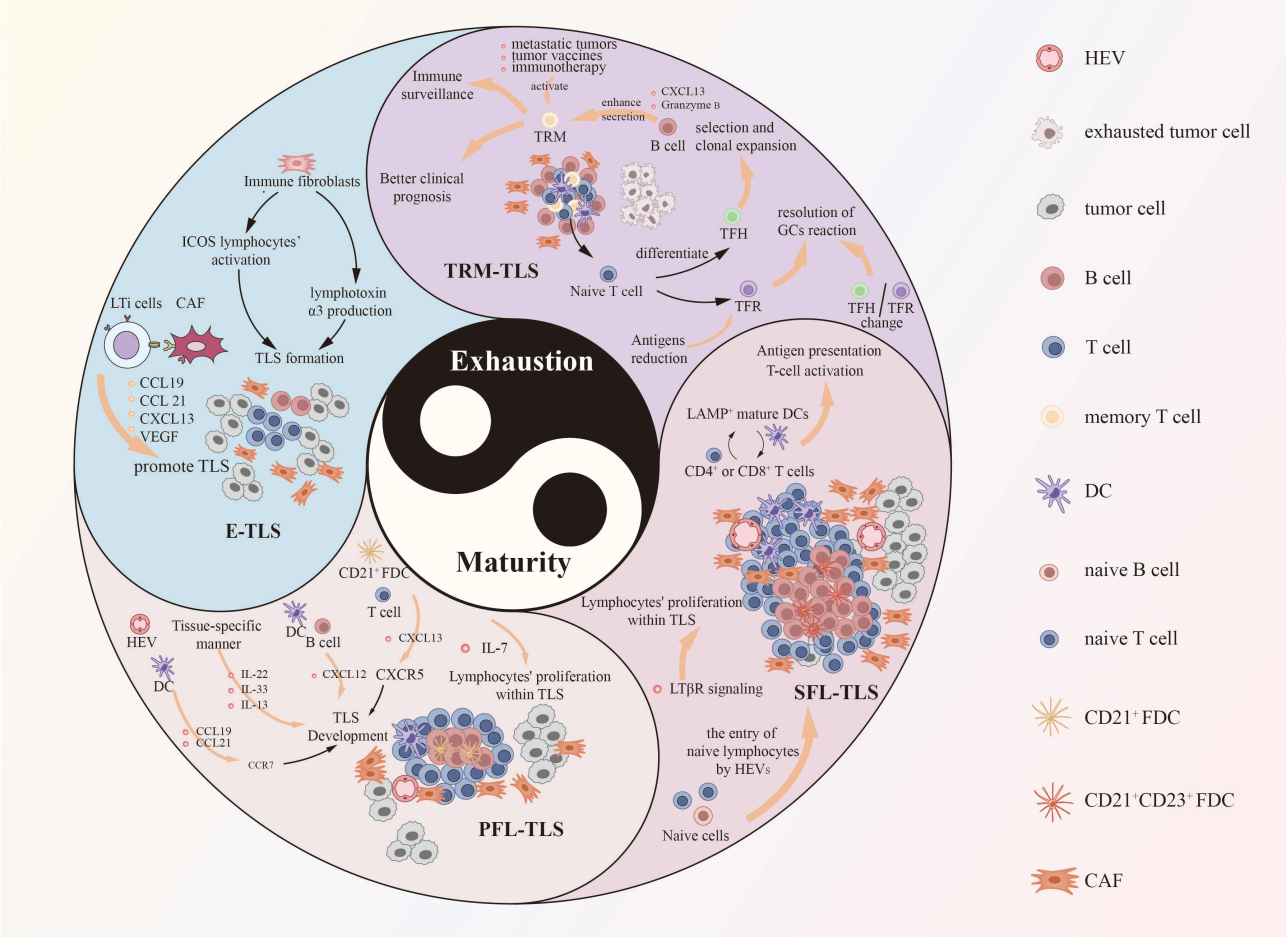
Life cycle of TLS. TLS can be classified as early TLS (E-TLS), primary follicle-like TLS (PFL-TLS) and secondary follicle-like TLS (SFL-TLS). Recently, a new TLS morphology has been identified. It may represent the resident memory stage of TLS. HEV, high endothelial venules; DC, dendritic cells; FDC, follicular dendritic cells; CAF, cancer-associated fibroblast; E-TLS, early TLS; PFL-TLS, primary follicle-like TLS; SFL-TLS, secondary follicle-like TLS; TRM-TLS, tissue-resident memory TLS; GCs, germinal centers; TFH, T follicular helper; TFR, follicular regulatory T.

TLS can be found at three distinct developmental stages (see **Figure 2**): early TLS (E-TLS), primary follicle-like TLS (PFL-TLS) and secondary follicle-like TLS (SFL-TLS). E-TLS represents the immature initial stage and is characterized by diffuse clusters of T and B cells without distinct segregation into T and B cell zones. While CD20+ B cell clusters are present, FDCs expressing CD21 and CD23 are absent. PFL-TLS, the second stage, is an organized collection of T and B cells containing FDCs, but lacking GC reactions. It is identified by the expression of CD20 and CD21, and the absence of CD23. Here, FDCs may facilitate antigen presentation to B cells. The mature and functional SFL-TLS stage is characterized by the presence of GCs, implying T and B cell interactions and B cell proliferation. It is identified by the expression of FDC signature (CD20/CD21/CD23) or GC signature (BCL6/Ki-67/AID/SEMA4A) (34).

Further development of TLS relies on HEVs expressing PNAds, which serve as entry pathways for naïve B and T cells. Despite collective efforts to define and standardize TLS states, the complexity of TLS and the TME hinders the establishment of a unified classification system. This developmental hierarchy highlights the dynamic nature of TLS formation and the importance of understanding its maturation process in the context of cancer immunity and therapeutic targeting.

Although few studies have investigated the sequential development of TLS from immature lymphoid aggregates to CD21^+^ primary follicle-like and mature CD21^+^CD23^+^ secondary follicle-like stages, indirect evidence suggests this temporal model of TLS maturation. This maturation process is likely driven by coordinated signaling through pathways such as lymphotoxin, CXCL13/CXCR5, and CCL19/CCL21/CCR7, although the specific contributions of individual chemokines at different stages remain unclear. Luther et al. cloned CXCL13 (40), CCL19, CCL21, and CXCL12 (41) into the rat insulin promoter and expressed them in pancreatic islets. These demonstrated that expression of the individual chemokines resulted in the formation of lymphoid aggregates of varying compositions and sizes. Ectopic expression of CCL19 resulted in small infiltrates composed of lymphocytes, DCs, HEVs, and stromal cells, while ectopic expression of CXCL12 induced infiltrates with few T cells but abundant DCs, B cells, and plasma cells.

Ectopic expression of CCL21, on the other hand, induced larger and more organized infiltrates than CCL19. Other chemokines, such as IL-22, IL-33, and IL-13 (17), may also contribute to TLS formation in a tissue-specific manner. Additionally, the source of individual chemokines may vary with the stage of maturation. A recent study on TLS in high-grade serous ovarian cancer revealed that CXCL13 production shifts from CD4^+^ T cells to CD21^+^ FDCs as TLS matures (42).

From a clinical perspective, the value of identifying all three stages in routine pathological assessment remains uncertain. Several studies have reported a discrepancy in patient prognosis between tumors exhibiting high TLS density and those exhibiting low TLS density, irrespective of the differing stages of TLS maturation (43, 44).

#### 3.2.3 TLS maturation

Mature GCs-containing TLS have been shown to be structurally and functionally similar to SLO, and to function as cellular immune hubs. In this capacity, they provide a communication platform and bridge between B cells and T cells in cancer patients. The aggregation of CD20^+^ B cells with surrounding CD3^+^ T cells is the primary feature that identifies and characterizes TLS. In contrast to SLO, which exhibits a homogeneous structure and function, TLS possesses a multifaceted tissue composition and cellular arrangement. The differential compositions of TLS may influence their function in pancreatic cancer.

In mature TLS (mTLS), the T-cell zone is supported by a stromal-derived FRCs network and serves as a site for antigen presentation and T-cell activation occurs due to interactions between LAMP^+^ mature DCs and CD4^+^ or CD8^+^ T cells. Within the B-cell zone, B cells undergo rapid proliferation, somatic hypermutation, and class switch recombination, which are processes associated with affinity maturation of antibody and the formation of memory B cells (26, 45). In the SLO, the GCs are distinguished by the presence of a dark zone, which is characterized by a proliferation of Ki67^+^ B cells, and a light zone where B cells undergo antibody affinity-driven selection (46). However, this distinct zonation is less commonly observed in TLS. Ruffin et al. (45) identified tumor-infiltrating B cells (TIL-B) in patients that exhibited GCs transcriptional signatures and spatially organized within TLS; both of which correlate with favorable clinical outcomes. Therefore, tumor-specific IgG and IgA antibodies can bind to Fc receptors on NK cells and macrophages, triggering antibody-dependent cellular cytotoxicity (ADCC) to kill cancer cells (47).

The presence of HEVs structures is a hallmark of mTLS (33), thereby facilitating the entry of naive lymphocytes into inflammatory sites (25). In SLO, LTβR signaling has been demonstrated to promote the growth and differentiation of HEVs, which are considered a defining feature of TLS.

In response to inflammation, HEVs undergo a series of morphological changes, transitioning from a flat, thin-walled structure to a mature cuboidal form (48, 49). The proliferation of lymphocyte beds within TLS is characterized by a bimodal process, whereby the initial phase is driven by IL-7, while the subsequent phase is facilitated by LTβR signaling, coinciding with the establishment of TLS (50).

#### 3.2.4 Tissue-resident memory TLS

The classical maturation model of TLS describes a linear development trajectory from E-TLS to TLS formed by mature follicular dendritic cell network (such as PFL-TLS and SFL-TLS) (1). However, based on the existence of “involuted” TLS identified by Shu et al. (51), we speculate that there may be more complex patterns in the TLS life cycle. Despite the progress that has been made in understanding the maturation stages of TLS, the tissue-resident memory phase of the TLS life cycle remains poorly characterized (**Figure 2**).

The mechanisms governing the resolution of GC within TLS remain an area of active investigation. Several lines of evidence from murine models suggest that the contraction phase of GC is not merely a passive consequence of antigen depletion, but an actively regulated process involving a shift in T cell subset equilibrium. For instance, while the lack of antigen or T cell help can lead to premature GC termination, recent studies highlight the role of regulatory T cells in orchestrating this process under physiological conditions (52). Jacobsen observed a sharp increase in T cells preceding GC contraction post-immunization, indicating that T cells may actively drive the resolution stage (53). This is complemented by functional studies showing that ectopic expression of Foxp3 in TFH cells is sufficient to reduce GC size, suggesting that T follicular regulatory (TFR) cells play a key role in limiting the GC reaction.

The dynamic balance between TFH and TFR appears to be critical in this context. Merkenschlager et al. demonstrated that during the GC response, newly recruited naive T cells continuously differentiate into both TFH and TFR cells (54). While TFH cells are known to promote B cell selection and clonal expansion during the initial phases, the emergence of TFR during the contraction phase, potentially driven by waning antigen availability, contributes to GC resolution. This shift in the TFH/TFR ratio may also explain observations in pathological states. For example, the disappearance of T/B cell segregation in TLS within the pancreas of the non-obese diabetic mouse model following the destruction of Langerhans β-cells may reflect a collapse of the supportive TFH infrastructure (55). Conversely, in human lung cancer with EGFR mutations, a decrease in TFH-like cells and subsequent impairment of TFH-B-TRM cooperation was linked to dysfunctional TLS formation and poor response to anti-PD-1 therapy (56). Taken together, these findings across both murine models and human tumors suggest that the lifecycle of TLS, from neogenesis to resolution, is governed by the dynamic equilibrium of TFH and TFR cells, and that disruption of this balance can lead to either premature TLS involution or impaired anti-tumor immunity.

Recently, Shu et al. (51) identified a novel morphology of TLS in the tumor regression areas of hepatocellular carcinoma (HCC) patients who underwent neoadjuvant immunotherapy prior to surgical resection. They reported “involuted” TLS morphology based on their location, morphology, and immune composition, which may potentially represent the terminal stage of the TLS life cycle. These “involuted” TLS are characterized by dispersed B cell follicles and a T cell zone enriched with sustained antigen presentation and T cell-mature DCs interactions. Moreover, these “involuted” TLS exhibit increased expression of TRM cell markers and expansion of CD8^+^ cytotoxic and tissue-resident memory clonotypes. The same situation may occur in pancreatic cancer. Furthermore, Hu et al. (57) reported that the presence of TLS and TRM cells in tumor tissues favored a superior response to anti-PD-1 therapy in patients with gastric cancer. They identified that activated B cells enhanced CXCL13 and granzyme B secretion by TRM cells, further revealing a crucial role for cellular communication between TLS-associated B cells and TRM cells in antitumor immunity. TRM cells often express elevated levels of inhibitory receptors such as PD-1 and TIGIT in TME, leading to an exhaustion state (58). However, proactive exhaustion is not always a bad thing. TRM cells exist in this newly discovered TLS structure and can be activated by signals such as metastatic tumors (59), tumor vaccines (60), immune checkpoint blockade immunotherapy (61), and exert immune surveillance. TRM cells may also fail to produce sufficient levels of these cytokines when dysfunctional, weakening the immune response against tumors (62). They may also alter interactions with other immune cells, such as DCs and macrophages, leading to impaired recruitment and activation of other immune cells that contribute to antitumor immunity (63, 64).

These may constitute tissue-resident memory TLS (TRM-TLS) that respond to future encounters with tumor. Further research is required to define the tissue-resident memory phase of TLS, to characterize the cellular and molecular changes occurring during this stage, and to elucidate its functional significance in cancer immunity and therapy.

### 3.3 Identification and detection of TLS

Investigation of TLS in pancreatic cancer tissues has paved the way for novel immunotherapeutic approaches. However, this progress underscores the urgent need for standardized protocols for the detection and quantification of TLS (65). To date, there is a lack of universally standardized criteria for the evaluation of TLS in cases of pancreatic cancer (33). The heterogeneity of tumor-associated TLS in pancreatic cancer is multifaceted, encompassing variability in cellular composition, structural organization, degree of maturation, and immunological function. Furthermore, TLS may be situated in diverse anatomical compartments, including intratumoral, peritumoral, and stromal regions, each with potentially unique microenvironmental conditions and immune cell profiles (66). This pronounced heterogeneity directly impedes the development of unified and reproducible criteria for TLS identification and classification. Furthermore, the interpretation of TLS-related findings is rendered more complex, thus hindering their integration into clinical value for pancreatic cancer.

Currently, several methods have been advanced for the detection of TLS. Conventional hematoxylin and eosin (H&E) staining is a widely employed technique for visualizing cellular aggregates. However, the identification of TLS can prove challenging in the absence of the expertise of trained pathologists. Immunohistochemistry (IHC) or immunofluorescence (IF) can reveal the cellular composition and organization of TLS. mTLS identification can be achieved in standard pathology laboratories using a combination of CD20, CD3, and CD23 markers with multiplex bright-field IHC and multiplex IF analyses detecting the hallmarks of mTLS, namely, the FDC network in GCs (43, 67).

In TLS, the selection of B cell markers is not static, but should be dynamically considered according to its life cycle. However, as a component of B cell antigen receptor, CD79a is expressed throughout the whole B cell differentiation stage from pro-B cells to plasma cells, and is a reliable marker for identifying the B cell lineage (68). In the PFL-TLS, CD79a helps to comprehensively evaluate the infiltration and aggregation of B cells (69). The structure and function of TLS reached the peak when TLS matured and formed the germinal center of CD20+CD21+CD23+. Therefore, combining CD20 and CD79a with plasma cell markers (such as CD138) can build a complete picture from the formation, maturation and function of TLS, which is essential for understanding the anti-tumor immune cycle driven by TLS.

Although traditional IHC and IF can identify the existence of TLS, it cannot fully analyze its transcriptome characteristics and its interaction with the surrounding microenvironment. Recently, Sidiropoulos et al. used spatial transcriptome technology to achieve high-resolution analysis of TLS niche in pancreatic cancer for the first time (70). Through unsupervised learning, they identified the TLS specific spatial gene expression program, and found that the maturation status, cell composition (such as B cell differentiation) of TLS was closely related to its spatial location in tumors, which provided a new perspective for understanding the heterogeneity of TLS in pancreatic cancer. Artificial intelligence (AI) has further facilitated the automation of image analysis, enabling the identification and quantification of TLS with greater accuracy. AI-driven deep learning algorithms have demonstrated considerable promise in early tumor screening, diagnosis, and prognosis across a wide range of malignancies (65). To date, optimal markers for accurate characterization of TLS remain elusive. The integration of TLS-specific biomarkers, advanced imaging modalities, and AI-driven analytical approaches holds significant potential for enhancing the detection (71) and quantitative assessment (72, 73) of TLS. Notwithstanding, this field remains the subject of ongoing investigation. A comprehensive overview of the most employed TLS detection methods, along with a summary of their respective features was summarized in **Table 2**.

**Table 2:** Summary of TLS detection methods.

Detection method	Advantage	Disadvantage	References
H&E staining	The morphology of TLS was detected in tumor slices fixed in formalin and embedded in paraffin Low cost, easy to execute	The extracted information was limited and did not provide any information about cells crosstalk in the TME	(65)
Multiple immunofluorescence: mIHC and mIF	Can conduct more in-depth research on the biology of TME, such as intracellular interactions Compared with H&E staining, analyzing multiple markers simultaneously provides a more accurate assessment of cell distribution	The presence of TLS was only indirectly determined	(149)
12-chemokine signature analysis	Quantitatively detect TLS at the gene level by evaluating the gene expression of 12 specific chemokines	Define the existence of TLS indirectly	(65, 150)
flow cytometry	Need single cell suspension of tumor tissue	Because the cell populations from TLS and non TLS regions are evenly mixed, it is difficult to visually detect the presence of TLS	(142)
Single-cell and spatial transcriptomic analyses	Clarify the location relationship between TLS and adjacent regions inspect the related cells crosstalk	High cost, difficult to popularize; Tumor samples from specific anatomical regions need to be analyzed	(71)
TLS 3D imaging	Provide information about the structure, volume and quantity of TLS, and even the interactions between cells in TLS	High cost, difficult to popularize; Tumor samples from specific anatomical regions need to be analyzed	(65)
AI (Artificial Intelligence) and Machine Learning Recognition Models	Automatically identify target cells within tumors Classify based on the morphological characteristics and tissue structure of immune cell subpopulations	No unified standard or model yet	(65, 151)

Achieving a precise characterization of TLS subtypes has the potential to provide significant benefits, facilitating personalized immunotherapy approaches and enabling the stratification of patients with various diseases. Furthermore, it offers the potential to identify biomarkers that can be used to predict disease prognosis and treatment responses. In addition, it permits the customization of therapies based on TLS subtypes to improve the treatment outcomes.

### 3.4 Relationship between TLS and pancreatic cancer prognosis

Over the past decade, several studies highlighted the role of TLS in regulating adaptive antitumor immune responses. As specialized sites for B-cell and T-cell activation, TLS are associated with improved survival rates in various solid tumors, including pancreatic cancer. Hiraoka et al. (66) found that the presence of intratumoral TLS was associated with a favorable patient prognosis in pancreatic cancer. Tumors with intratumoral TLS exhibited higher infiltration of T and B cells, lower infiltration of immunosuppressive cells, and increased expression of Th1 and Th17-related genes. Alternatively, Gunderson et al. (74) confirmed that mTLS showed higher rates of B-cell somatic hypermutation, suggesting that high-quality neoantigens promote durable immune memory and improve survival in PDAC patients. This study linked GC reactions in associate with neoantigen burden, humoral immunity and long-term survivorship in pancreatic cancer. Kinker (20) also found that GC signatures in TLS were associated with improved survival in PDAC.

The findings of an omics study (75) on the neural invasion (NI) of pancreatic cancer suggest that low-NI tumor tissues were enriched in TLS, which co-localized with non-invasive nerves. In contrast, high NI tissues exhibited NLRP3^+^ macrophages and cancer-associated myofibroblasts surrounding invaded nerves. They also identified a unique intraneural NRP2^+^ fibroblast population and three distinct Schwann cell subtypes. TGFBI^+^ Schwann cells, located at the leading edge of NI, were induced by transforming growth factor-beta (TGF-β) signaling, facilitated tumor cell migration, and were associated with poorer survival.

TA-TLS can be located either within the tumor (intratumoral) or at the tumor periphery (peritumoral) (76). It is worthy of note that lower levels of PDAC-containing intratumoral TLS are observed in comparison to other solid tumor types (66), which has constrained the qualitative and quantitative analysis of their spatial organization and cellular composition. Furthermore, there is currently no unified analytical model in clinical practice to predict PDAC patient survival based on TLS or intratumoral TLS.

The structure and maturity of TLS govern its functional efficacy and predict clinical results. Vanhersecke et al. (77) conducted a large-scale retrospective analysis of three cohorts of cancer patients treated with anti-PD1/PD-L1 antibodies and reported that the presence of intratumoral mTLS was associated with longer overall survival in various solid tumors, including PDAC. The presence of TLS was closely correlated with improved T-cell infiltration and activation (78, 79). Consequently, it is hypothesized that the presence of TLS within tumors may serve as a predictive biomarker for favorable responses to therapy involving immune checkpoint inhibitors (80).

The prognostic value of the main constituent cell of TLS, CD20^+^ B cells, in PDAC has produced inconsistent results. Rupp (81) found that an increased frequency of proliferating CD20^+^Ki67^+^ B cells was correlated with longer overall survival in patients with PDAC. It was also confirmed by several other studies (82, 83), while Wang (84) found that TLS failed to predict recurrence and survival in patients after surgery for PDAC. The meta-analysis incorporating numerous studies revealed no correlation between B-cell frequency and survival in patients with PDAC (85). The discordant findings may be attributable to the presence of CD20^+^Ki67^+^ B cells in more than just mTLS. The presence of actively replicating B cells may indicate a restorative immune microenvironment and an active adaptive anti-tumor immune response following treatment. On one hand, TLS can enhance immune surveillance and promote anti-tumor immunity (86), while on the other hand, they may exert tumor-promoting effects (87–89). Although many studies suggest a positive prognostic value for TLS, consensus is lacking with respect to its role in certain cancers.

Chronic immune activation, a hallmark of both autoimmune diseases and cancer, can paradoxically lead to T cell exhaustion. In chronic autoimmune conditions such as lupus nephritis (90, 91), persistent self-antigen stimulation creates a chronically inflamed microenvironment that drives the exhaustion of infiltrating T cells, including potential TRM cells. We propose that a similar mechanism operates in the tumor context. TLS and associated TRM-TLS may serve as a persistent source of immune activation against tumor antigens. While this can initially control tumor growth, the sustained antigen exposure within the TME, similar to the autoimmune setting, may eventually drive tumor-infiltrating lymphocytes into a state of exhaustion. This dual role provides a mechanistic basis for the biphasic clinical phenomenon observed in some patients, such as those with pancreatic cancer, where an initial response to therapy is followed by relapse (92).

This biphasic effect, initial efficacy followed by subsequent failure, is conceptually analogous to the chronic, relapsing-remitting course of many autoimmune diseases. In both scenarios, the very mechanism designed to combat the disease (immune activation) eventually contributes to its persistence or progression through the induction of exhaustion. For example, in the phase 1 clinical trial of CAR-T cells in pancreatic cancer (92), the initial antitumor activity was limited by the rapid onset of T cell exhaustion, mirroring the dysfunctional state seen in chronic autoimmune lesions. Therefore, inhibiting aberrantly activated TLS or reversing the associated T cell exhaustion (93) might represent a novel strategy to restore immune function and improve patient prognosis, effectively breaking this detrimental cycle. It is also speculated that tumor evolution and heterogeneity (94) may contribute to this phenomenon, wherein the molecular phenotype of the tumor evolves during progression, and a timely response by TRM-TLS may suppress tumorigenesis and development. However, a temporal discrepancy between the rate at which TRM-TLS recognizes relevant molecular phenotypic changes and the pace of molecular phenotypic variation in tumors such as pancreatic cancer may contribute to the dual role of TLS in immune regulation and facilitate immune escape.

These inconsistent findings suggest that the phenotype and function of TLS may be dependent on its developmental state, cellular composition, and distance from the tumor tissue.

## 4 Antitumor therapy and TLS for pancreatic cancer

### 4.1 TLS and chemotherapy synergetic relationship

The mechanism of TLS formation involves intrinsic and extrinsic factors associated with tumor cells. Tumor-derived neoantigens are key drivers. They stimulate cytokine signaling pathways and recruit immune cells, thereby facilitating TLS development within the TME (95, 96). Tumors with high TLS and low tissue-resident exhausted CD8^+^ T cells have superior clinical outcomes with nivolumab (97). The impact of chemotherapy on TLS evolution remains complex and partially unresolved. On one hand, chemotherapy promotes the release of neoantigens that can activate cytokine pathways and enhance immune cell function to potentiate anti-tumor immunity. On the other hand, if immune reconstitution after chemotherapy is insufficient or delayed, adverse patient outcomes may ensue. For example, the chemotherapeutic drug paclitaxel counteracts immune checkpoints to block immune cell expansion (98). These results provide a scientific rationale for considering TLS induction as a therapeutic approach. They underscore the potential for TLS to enhance the anti-tumor activity of chemotherapy in PDAC and inform the development of more tailored immuno-oncological strategies.

Hepatic arterial infusion chemotherapy (HAIC) significantly enhanced the formation of TLS in HCC tissues, associated with improved treatment response and prolonged progression-free survival in patients (99). Mechanistically, HAIC induced lymphotoxin beta (LTβ)-expressing central memory CD4^+^ T cells. These cells activated MMP2^+^ fibroblasts and FOLR2^+^CCL4^+^ macrophages via the LTβ–LTβR axis, thereby promoting TLS development. Furthermore, the CXCL12-CXCR4 axis served as a key mediator to facilitate the recruitment of these cells to tumors, consequently fostering TLS formation and enhancing anti-tumor immunity. Following HAIC in HCC, there was an increase in CD4^+^ T cells, CD20^+^ B cells, and DCs subsets, accompanied by enhanced intercellular communication (99). HAIC also promoted TLS formation, with moderately exhausted PD-1^+^CD8^+^ T cells that exhibited an anti-tumor phenotype accumulating preferentially near these structures (100).

### 4.2 TLS is associated with the efficacy of immune checkpoint inhibitors

Among cancer therapies, ICI has emerged as a prominent approach, substantially enhancing anti-tumor immune responses. ICI targets T cell negative regulatory proteins, such as programmed death-1 protein (PD-1) and cytotoxic T lymphocyte antigen-4 (CTLA-4) (100). However, the efficacy of ICI is often limited in the absence of a pre-existing immune response within the TME. Thus, identifying reliable response biomarkers is an urgent medical need. TLS, as the sites for generating circulating effector memory cells that control tumor recurrence, provide a unique opportunity to guide the next generation of clinical trials in the expanding field of immuno-oncology.

Several recent studies have provided evidence for the predictive value of TLS for ICI. The presence of TLS and active B cell infiltrates in the pre-treatment biopsies of melanoma, renal cell carcinoma, soft tissue sarcoma, and urothelial carcinoma (UC) has been demonstrated to correlate with the response to PD-1 inhibition or the combination of PD-1 and CTLA-4 (101). Similarly, pre-treatment biopsies from responders to melanoma ICI contain many TLS components. In a checkpoint blockade-resistant tumor model in mice, combination therapies capable of inducing TLS-sensitive tumors to ICI and induced the production of effector and memory T cells, implying that TLS creates a TME that allows ICI to be effective (102). The study (NCT04144608) enrolled 40 treatment-naive patients with initially unresectable stage IIIA-IIIB NSCLC, which showed that the TLS status of the patients was associated with the induction of immunotherapy responses (103). Jiao et al. (104) revealed that acetyl-CoA acetyltransferase 1 (ACAT1) limited the efficacy of ICI in NSCLC by impeding TLS in the TME. Targeting ACAT1 in tumor cells reduced mitochondrial hypersuccinylation and oxidative stress, enhancing TLS abundance and improving the efficacy of ICIs in preclinical murine models of NSCLC.

Meanwhile, analysis of tumor biopsies revealed that ICI treatment also promotes TLS formation. In patients with high-risk melanoma (105), non-small cell lung cancer (106), bladder cancer (107) and PDAC (70) undergoing neoadjuvant ICI therapy, those who responded well showed an enrichment of TLS-related B cells. Thus, ICI therapy has been demonstrated to increase the number and size of TLS, thereby contributing to tumor control. Combined with ICI therapy, hierarchical protein nano-crystalline hydrogel with extracellular vesicles also induces the TLS within the tumor, promoting immune responses against ICI-resistant tumor (108). Interferon-responsive HEVs secreting CXCL9 facilitates the recruitment of CXCR3^+^CD4^+^ T cells into TLS, which is strongly associated with prolonged survival and enhanced response to ICI (109). The intracranial TME of patients with NSCLC after immunotherapy with anti-PD-1 mAb or anti-CTLA4 mAb featured TFH and B cells infiltration as TLS (110).

### 4.3 Preoperative NACT interacts with TLS

In the past decade, survival in patients with BRPC or LAPC has improved with the use of preoperative NACT regimens. The NACT group demonstrated favorable prognoses in comparison with the upfront surgery group, as evidenced by the analysis of 140 pancreatic cancer patients (111). Tanaka et al. (112) found that the presence of TLS was associated with longer survival in pancreatic cancer, as well as high levels of CD4^+^ TILs, CD8^+^ TILs and CD45RO^+^ TILs. These data supported the surrogacy of TLS for vigorous anti-tumor immune response characterized by high levels of helper and cytotoxic T cells and their prognostic role.

NACT helps maintain intratumoral T cell phenotypes sensitive to ICI and facilitates the formation of TLS, thereby promoting a “hot” TME in high grade serous ovarian cancer (HGSOC) lesions (113). The increases of the density of intratumoral TCF1^+^CD8^+^ T cells significantly improved survival in syngeneic models with high tumor mutational burden when combined with PD-1-targeted ICI. A single-arm, phase II trial (ChiCTR2200066119) (114) tested 2 cycles of neoadjuvant immunochemotherapy (NAIC) with camrelizumab, nab-paclitaxel, and cisplatin in locally advanced oral squamous cell carcinoma patients. Exploratory analysis showed that patients with major pathological response (MPR) exhibited a higher density of baseline CD4_Tfh_CXCL13 cells and an increased density of TLS after NAIC.

In another research, a question has emerged concerning the impact of NACT on the pancreatic cancer TME. Lutz et al. (115) investigated the effects of a granulocyte-macrophage colony-stimulating factor (GM-CSF)-secreting, allogeneic PDAC vaccine (GVAX) given alone or in combination with low-dose cyclophosphamide, and found that treatment with GVAX transformed “nonimmunogenic” tumors into ‘immunogenic’ tumors, promoting T-cell infiltration and the formation of TLS. Recently, Mota Reyes et al. (116) analyzed the effect of NACT on the TME in 37 patients with locally advanced PDAC and found that NACT remodeled the pancreatic cancer microenvironment by selectively depleting regulatory T cells (Tregs) and myeloid-derived suppressor cells (MDSCs). This remodeling enhanced the infiltration of anti-tumor immune cells while reducing stromal activation and neural invasion. In a separate study (117), PDAC patients who underwent upfront surgical resection or who received neoadjuvant FOLFIRINOX with or without neoadjuvant radiotherapy followed by surgical resection were selected for study. The significant alterations in the expression and/or spatial distribution of immunologically relevant proteins in different regions (tumor cell rich, immune cell rich, stromal cell rich) of the TME were identified. These studies suggest that NACT induces the release of new antigens that promote immune activation in PDAC, making them beneficial for anti-tumor immunity in this context.

The extent to which all patients with PDAC derive immunological benefit from preoperative NACT remains unsettled, particularly in relation to the induction and maintenance of anti-tumor immunity (116–118). Rupp et al. (81) observed that NACT was associated with a marked reduction in B cells, plasma cells, and TLS, accompanied by downregulation of key B cell signaling pathways. Their data further indicated that the subset of CD38^+^CD138^+^ plasma cells infiltrating PDAC was diminished following NACT, with these cells were predominantly situated outside TLS structures. In the primary resection group, higher frequencies of plasma cells, especially clusters in close proximity to TLS, were observed. This finding suggests a potential role for TLS in promoting plasma cell differentiation within PDAC. In contrast, Kuwabara et al. (111) documented a reduction in the total area occupied by TLS in NACT treated PDAC, Zou (15) presented evidence indicating that TLS in the NACT group exhibited reduced numbers, diminished size, and indications of immature development. These divergent findings underscore substantial heterogeneity in the observed effects of NACT on TLS prevalence, maturation, and associated immune cell populations in PDAC. Collectively, such inconsistencies highlight the necessity for further systematic investigations to determine the precise immunomodulatory outcomes of NACT and to clarify its impact on TLS in PDAC patients.

## 5 Prospects for therapeutic induction of TLS

**Figure 3:**
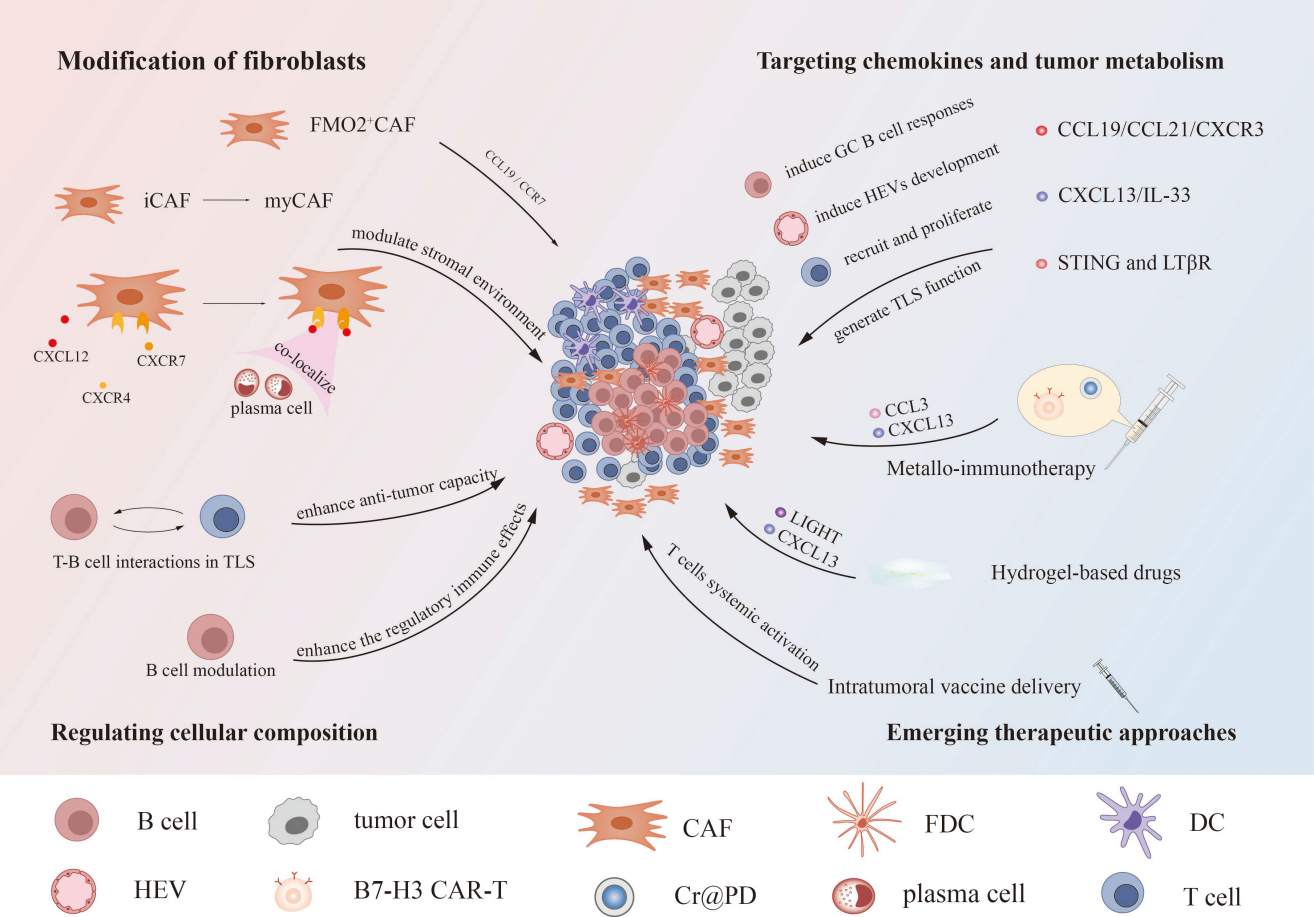
Prospects of therapeutic induction of TLS. Pancreatic cancer patients have a poor response to immunotherapy alone. Therapeutic induction of TLS to enhance anti-tumor immune function is one of the promising research directions. The following list details several possible strategies for therapeutic induction of TLS. In the stromal cell-rich immune microenvironment of pancreatic cancer, fibroblasts are one of the most dominant cells. TLS generation or enhancement of TLS immune function can be induced by modifying fibroblasts. The expression of relevant chemokines which induce TLS generation can also be a therapeutic induction strategy. The TLS immune function of can be enhanced through modifications in the composition of TLS. In addition to the several therapeutic directions previously described, promising strategies for the therapeutic induction of TLS have emerged in recent years, including metalloimmunotherapy, hydrogel-based drugs, and intratumoral vaccine delivery. HEV, high endothelial venules; DC, dendritic cells; FDC, follicular dendritic cells; CAF, cancer-associated fibroblast; apCAF, antigen-presenting CAFs; STING, stimulator of interferon genes; LTβR, lymphotoxin-β receptor.

The immunosuppression within the TME, characteristic of pancreatic cancer, often limits the efficacy of immunotherapy. The potential to induce TLS and thereby remodel the TME to enhance tumor destruction by immune cells is, therefore, a compelling prospect. The crucial question is how to design TLS to avoid an uncontrolled autoimmune response. In this review, we propose several strategies that could facilitate the achievement of this objective (**Figure 3**).

### 5.1 Modification of fibroblasts

In a recent study, Ashina et al. (119) investigated the relationship between tumor-stromal collagen, molecular and immune characteristics, and tumor progression in 169 patients with PDAC after surgery. They found that tumors with low collagen levels had less TLS, poorer differentiation and shorter overall survival. The antigen-presenting CAFs (apCAF), characterized by high MHC II expression were preferentially located near TLS in gastric cancer (120). In another study, FMO2^+^CAFs increased anti-PD-1 treatment efficacy by promoting TLS formation and increasing the infiltration of CD8^+^T cells and M1-like macrophages through the C-C motif chemokine ligand 19 (CCL19) and C-C motif chemokine receptor 7 axis (121).

In PDAC, CAFs, a key cell type in the stroma, produce abundant extracellular matrix and exhibit crosstalk with cancer cells inducing chemoresistance (122). Fibroblasts can be engineered to produce lymphopoietic factors and chemokines and then implanted or directed to the tumor site.

It may be hypothesized that the resistance to various therapeutic modalities in PDAC emanates from enhancement of immunity. The potential of CAFs of the extracellular matrix as immunotherapeutic targets to modulate the stromal environment of PDAC by converting tumor-promoting (αSMA-low IL-6-high inflammatory CAFs; iCAFs) to tumor-suppressing (αSMA-high IL-6-low myofibroblasts; myCAFs) may enhance immunity (123).

The plasma cell aggregates in PDAC tended to co-localize with fibroblasts expressing chemokine receptor CXCR4 in the vicinity of the TLS. As suggested in some studies (124, 125), these fibroblasts may resemble mesenchymal stromal cell-like fibroblasts or follicular reticulocytes in the bone marrow, which have been demonstrated to promote the survival of early and memory plasma cells. Upon binding to the cognate receptors CXCR4 and CXCR7 (ACKR3), the ligand-receptor complex is internalized, and CXCR2 is transported for lysosomal degradation (126). This finding lends further support to the hypothesis that the TME may be enhanced by modulating CXCL12^+^ fibroblast-mediated plasma cell migration near the TLS of PDAC, thereby improving patient prognosis. Kinker et al. (20) found that tumor-reactive T cells exposed to TGF-β produce the B cell chemokine CXCL13, which may drive TLS formation.

It has been observed that CAFs exhibit both intratumoral and intertumoral heterogeneity, characterized by a high degree of plasticity, with the presence of at least four well-defined subtypes (127). In 2025, Rui You (128) performed single-cell and spatial transcriptomic analyses on 39 tumors from 24 patients in order to reveal the mechanisms of radiotherapy resistance and immune evasion in recurrent nasopharyngeal carcinoma (rNPC). They demonstrated a significant enrichment of MCAM^+^ CAF in rNPC, which promoted radioresistance via the collagen IV–ITGA2–FAK–AKT signaling pathway. Collagen IV was found to inhibit T cell infiltration, and CD8^+^ ZNF683 cells exhibited reduced cytotoxicity. The abundance of B cells and TLS significantly diminished in rNPC. These differences in chemokine expression can promote spatial immune compartmentalization within individual tumors, and may also play a key role in intertumoral (CAF and immune cells) crosstalk, an aspect that can be targeted in therapy (129).

### 5.2 Targeting chemokines

The expression of the chemokines, namely, CCL19, CCL21, and CXCR3 has been shown to induce the formation of TLS. For instance, the forced expression of CCL19, CCL21, or CXCR3 in normal pancreatic β-cells has been shown to result in the formation of pancreatic toxin-signaling TLS (40). While the role of the chemokine CXCL13 is evident in the initial phase of TLS formation, its contribution to TLS organization in conjunction with GCs formation is not apparent. This observation serves to emphasize the notion that TLS development is the result of the orchestration of multiple factors (42). IL-33 has been demonstrated to “mobilize” intestinal group 2 innate lymphoid cells (ILC2s) to pancreatic tumors and cooperate with myeloid cells to organize the generation of TLS, in order to inhibit pancreatic tumor growth by promoting TLS production and enhancing TLS activity (17). Lineage tracing was used to fate map naïve T cells and follow their differentiation into TFH and TFR during an ongoing GC reaction (54). In the early GC, newly recruited T cells develop into TFH, whereas cells entering during the contraction phase develop into TFR cells that contribute to GC dissolution. Thus, invasion of ongoing GCs by newly developing TFH and TFR helps remodel the GC based on tumor neoantigen availability. Adenovirus-directed cytokine therapy (such as oncolytic adenovirus encoding hTNFα and hIL-2 and non-replicate adenoviruses encoding mTNFα and mIL-2) for the induction of TLS has been tried in other solid tumors (130). Moreover, the efficacy of this therapy was not exclusive to tumor region, it also led to an increase in CD4^+^ and CD8^+^ naïve cells and central memory cells in lymph nodes and spleen to activate the whole immune system.

The stimulator of interferon genes (STING) is an intracellular danger signal sensor responsible for the induction of type-I interferon (IFN)-stimulated genes, hence providing a crucial bridge between innate and adaptive immunity. Concurrent activation of STING and LTβR result in CD8^+^T cell-dependent tumor suppression while inducing HEVs development and germinal center B cell responses in tumors to generate TLS function in a T cell-dependent manner (131). Concurrent activation of STING and LTβR in neoadjuvant murine models (131) induces B cell response, promotes antitumor immunity, suppresses tumor recurrence, and improves long-term survival rates. This combination has been demonstrated to facilitate B cell expansion and maturation, enhances the recruitment and proliferation of both CD4^+^ and CD8^+^ T cells, thereby amplifying tumor-specific immune response.

Targeting tumor metabolism may promote the expansion of TLS, thereby exerting anti-tumor effects. ATP citrate lyase (ACLY) links substrate availability and mitochondrial metabolism with lipid biosynthesis and gene regulation. In the mouse model (132), genetic suppression of ACLY was found to correlate with increased expression of the chemokine CXCL13, elevated levels of tumor-infiltrating B cells, and enhanced TLS formation. The depletion of B cells served to nullify the anti-tumor effects of ACLY inhibition. The reprogramming of the TME by rAAV-Ccl3 was found to be a key factor in the promotion of immune cell recruitment and TLS formation, consequently leading to the suppression of tumor growth via immune engagement (133). These findings underscore the potential of targeting specific chemokine axes within TLS to remodel the tumor immune microenvironment and suppress cancer progression.

### 5.3 Regulating the cellular composition of TLS

The capacity for optimal T cell mediated suppression (TMS) can be ensured by manipulating the cellular components of the TME. Hindley et al. found that the removal of Tregs did not affect the tumor growth rate, although it promoted tumor-specific T cell activation (134). The elimination of Treg may encourage the development of tumor, which in turn increases lymphocyte infiltration and the destruction of tumor tissue (134). Joshi et al. (135) found that Tregs in TA-TLS inhibited anti-tumor T cell responses in a genetically engineered mouse model of lung adenocarcinoma. Tregs in TA-TLS may inhibit endogenous immune responses to tumors, and targeting of these cells may provide therapeutic benefit to cancer patients. Colbeck (136) found that removing Tregs in a 3-methylcholanthrene-induced tumor model induced CD8^+^ T cell activation and promoted HEVs formation within the tumor. These studies suggest that inhibition of Tregs results in a more robust TLS and enhanced anti-tumor immunity. Recently, Wang et al. (137) found that vitamin B6 in CD160^+^ CD8^+^ T cells from TLS tissue samples of stage 4 gastric cancer patients reduced the ubiquitination modification of HIF-1α by MDM2, which attenuated the degradation of HIF-1α, leading to the transcriptional up-regulation of CXCL13 expression, facilitating the recruitment of CXCR5^+^ B cells and the formation of TLS. This study demonstrates that T-B cell interactions in TLS enhance its anti-tumor capacity.

Many studies focus on T cell responses; however, mounting evidence (138) suggests that B cells may be integral to the establishment of an organized immune response, particularly through TLS. The mechanisms of B cell response are multifaceted, encompassing antibody-dependent cytotoxic effects and phagocytosis (139–142), the promotion of CD4^+^ and CD8^+^ T cell activation (143), and the maintenance of antitumor immune memory (105). Consequently, the enhancement of the regulatory immune effects of TLS by means of B cell function modulation should be explored as a treatment tool. The co-localization of B cells and DCs within the TLS was observed to facilitate intercellular communication, thereby promoting adaptive anti-tumor responses in a mouse model of pancreatic cancer (12). Ruffin (45) suggested that the exploration of therapeutic approaches to enhance TIL-B responses should be prioritized in future research, with a view to complementing current T-cell-mediated immune therapies. It is also possible to enhance the functional and immunological effects of TLS by augmenting the GCs B cell response. For instance, a recent study demonstrated that simultaneous activation of the STING and LTβR pathways could potentiate TLS formation by specifically augmenting the germinal center B cell response, leading to an improved anti-tumor immune response (131).

Recent study has utilized novel technologies to investigate the combined effects of T and B cells in amplifying the anti-tumor capabilities of TLS. Li et al. (144) found that intravesical lysosomal immunotherapy induced T cell infiltration and TLS formation, and that the formation, expansion, and maturation of TLS correlated with a complete pathological response. These findings suggest that synergistic humoral and cellular immune responses are important.

### 5.4 Other emerging therapeutic approaches

In addition to the aforementioned, there are some other promising strategies for the therapeutic induction of TLS that have emerged in recent years. These include metallo-immunotherapy, hydrogel-based drugs, and intertumoral vaccine delivery.

Metallo-immunotherapy is a strategy that has been shown to be effective in activating antitumor immunity. Recently, biodegradable polydopamine as a versatile carrier for the nanoparticles based on chromium nanoparticles (Cr NPs) for cancer photo-metallo-immunotherapy has been enabled synergistic CAR-T cell therapy (145). The trivalent Cr3^+^ ions, which are the major degradation product of these nanocomposites, can increase the CXCL13 and CCL3 chemokine expressions to generate TLS in the solid tumor tissues, facilitating the CAR-T cell infiltration.

Kuwentrai reported that a single injection of HHA-CPP⊂CB (8) hydrogel containing the CXCL13 chemokine and LIGHT cytokine effectively increases TLS density, facilitates mTLS formation, suppresses tumor growth, and extends survival in a B16-OVA melanoma mouse model (146). When combined with anti-PD1 immune checkpoint blockade therapy, it further enhanced tumor suppression and TLS formation, demonstrating its potential application value as a drug delivery platform in cancer immunotherapy. As a cutting-edge field of nanomedicine, covalent organic frameworks (COF)-based photosensitizers (147) have been extensively employed in cancer phototherapy in recent years. A Phase I study (NCT04059588) of intertumoral injection of 2141-V11 (148), an Fc-engineered anti-CD40 agonistic antibody with enhanced binding capacity to the inhibitory receptor FcγRIIB, was conducted in twelve metastatic cancer patients. It was found that 2141-V11 induced tumor regression, associating with systemic activation of CD8^+^ T cells and presence of mTLS in complete responders.

These emerging therapeutic approaches demonstrate the potential to promote the formation of TLS in practical applications, exhibiting complementary and synergistic effects. This not only introduces renewed dynamism into prevailing anticancer strategies but also underscores the feasibility of developing novel treatment modalities through the targeted modulation of TLS.

## 6 Conclusion and perspectives

In recent years, there has been a marked escalation in scholarly attention to the interplay between the lifecycle of tertiary lymphoid structures (TLS) and the immunotherapeutic strategies, particularly concerning their potential role in the antitumor response in pancreatic cancer. Clinical investigations have increasingly highlighted the prognostic significance of TLS and established those multiple therapies can potentiate TLS evolution, indicating a potential synergistic benefit. Nevertheless, the complexity of TLS biology presents several critical research gaps. Foremost among these is the limited mechanistic understanding of how specific components of TLS--such as distinct immune cell subsets, stromal elements, and their molecular interactions--contribute to either beneficial or adverse clinical outcomes. Furthermore, the temporal dynamics governing TLS induction, maturation, maintenance, and resolution in response to different therapeutic modalities remain poorly elucidated. Another significant area of uncertainty pertains to the precise regulatory pathways that modulate TLS function within the immunosuppressive tumor microenvironment of pancreatic cancer. Presently, there is no academic consensus regarding the efficacy of intervening in TLS evolution or function to improve patient prognosis. Addressing these gaps requires methodologically rigorous studies employing longitudinal analyses, advanced spatial and single-cell technologies, and well-defined preclinical models to establish causality and therapeutic utility. Continued investigation into these unresolved questions will be essential to advance the translational potential of TLS-targeted therapies.
